# Qualitative and quantitative research of medication review and drug-related problems in Hungarian community pharmacies: a pilot study

**DOI:** 10.1186/s12913-019-4114-1

**Published:** 2019-05-03

**Authors:** András Szilvay, Orsolya Somogyi, Attiláné Meskó, Romána Zelkó, Balázs Hankó

**Affiliations:** University Pharmacy Department of Pharmacy Administration, Semmelweis University, Hungary; 7-9 Hőgyes Endre street, Budapest, H-1092 Hungary

**Keywords:** Drug-related problem, Medication review, Pharmaceutical care, Community pharmacy, Vitamin K antagonist, ACE inhibitor

## Abstract

**Background:**

Pharmaceutical care is the pharmacist’s contribution to the care of individuals to optimize medicines use and improve health outcomes. The primary tool of pharmaceutical care is medication review. Defining and classifying Drug-Related Problems (DRPs) is an essential pillar of the medication review. Our objectives were to perform a pilot of medication review in Hungarian community pharmacies, a DRP classification was applied for the first time. Also, our goal was the qualitative and quantitative description of the discovered DRPs, and of the interventions for their solution in order to prove the safety relevance of the service and to map out the competence limits of GPs and community pharmacists to drug therapy.

**Methods:**

The project took place in Hungarian community pharmacies. The study was performed with patients taking vitamin K antagonist (VKA) and/or ACE inhibitor and NSAID simultaneously (ACEI-NSAID). 61 pharmacists and 606 patients participated in the project. Pharmacists reviewed the medication for 3 months and the classification of DRPs was performed (category of DRP1 – DRP6). Patient data were statistically analyzed.

**Results:**

Patients consumed on average 7.9 ± 3.1 medications and other products. 571 DRPs were detected in 540 patients, averaging 1.06 DRPs per patient (SD = 1.07). The highest frequency category was DRP5 (non-quantitative safety problem; 51.0%). The most common root cause was an interaction (42.0%) and non-adherence (19.4%.). The most commonly used intervention was education (25.4%) and medication replacement by the pharmacist (20.1%). The changing of the frequency and dosage in any direction were negligible.

**Conclusions:**

Patients are struggling with many DRPs that can be assessed and categorized by this system and which remain unrecognizable without pharmacists. Further projects need to be developed to assist in the development of physician-pharmacist cooperation and the widespread dissemination of pharmaceutical care.

## Background

According to the definition of Pharmaceutical Care Network Europe “Pharmaceutical Care is the pharmacist’s contribution to the care of individuals to optimize medicines use and improve health outcomes”. [1] The goal of the pharmacists is to collect the patient’s medications (Rx, OTC) and other products (e.g. dietary supplements) to ensure their necessity, efficacy and safety. [2]

The main tool of pharmaceutical care is medication review, “a structured, critical examination of a patient’s medicines with the objective of reaching an agreement with the patient about treatment, optimizing the impact of medicines, minimizing the number of medication-related problems and reducing waste”. [3] The best way to make medication review is in collaboration with the patients and their general practitioners. [4, 5] To demonstrate the benefits of medication review, several but controversial articles have published. It reduces the number of cases requiring emergency care [6, 7], the number of (re) hospitalizations, but its beneficial effects on quality of life, adverse drug reactions and mortality are non-significant in high-risk groups. [7, 8] However according to other articles it reduces the number of (unnecessary) drugs [9–12], it helps to detect and solve drug-related problems (DRPs) [9, 13–17], especially in collaboration with hospital pharmacists [18], it increases the patients’ trust in the therapy [19] and the cost-effectiveness of the treatment [20].

Defining and classifying drug-related problems is an essential pillar of the medication review. The drug-related problems are “situations in which in the process of use of medicines cause or may cause the appearance of a negative outcome associated with the medication.” [21] There are many reasons for the drug-related problems, which may result that drug therapy is not achieving its purpose or even becoming harmful. There are more than 20 types of DRP classification system in the literature, which differ in, e.g. DRP groups and methodology. [22] It is also important to involve patients in the process of detecting drug-related problems. [23]

The aim of our research was to perform a pilot of medication review in Hungarian community pharmacies as part of basic pharmaceutical care, using a drug-related problem classification for the first time to lay the foundation for wider adoption of this service in Hungary. Also, our goal was the qualitative and quantitative description of the discovered drug-related problems, and of the interventions for their solution in order to prove the safety relevance of the service and to map out the competence limits of GPs and community pharmacists to drug therapy. The latter can contribute to the development of a future “target model” of doctor-patient-pharmacist cooperation.

## Methods

### Description of the project

The project took place between December 2015 and August 2016. The data were collected by pharmacists (they have not received monetary compensation) participating in specialist training at Semmelweis University. The participation of pharmacists was obligatory to complete the second year of the training, and the cooperative pharmacies were their own workplaces. All participating pharmacies were accredited at the Semmelweis University. All the participating pharmacists from all around the country went to Budapest and participated in one-day training at Semmelweis University, which included the description and requirements of the project, and the presentation of the drug-related problem classification.

**Fig. 1:**
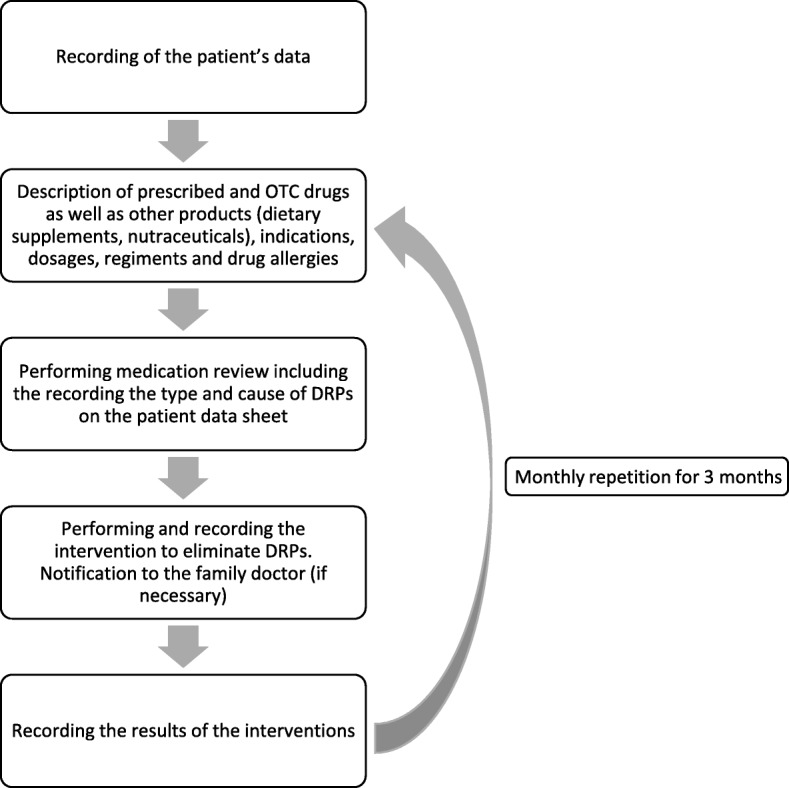
The process of the occasions

The pharmacists at the beginning of the project visited family practitioners working near the pharmacy, practically those whose patients often go to the pharmacy. They were invited to participate in the project. After that, the patients were involved in the pharmacy. The study was performed with patients taking vitamin K antagonist (VKA) and/or ACE inhibitor and NSAID simultaneously (ACEI-NSAID). The latter is considered as a high-risk group because of the increased chance of renal failure [24] and the potential inadequacy of the therapy [25]. A patient could have been in both categories. Patients had to be at least 18 years old, had to buy medicines themselves and had to be the patients of the general practitioner involved. All pharmacists tried to have around 10 patients. The process of the first and further occasions is described in Fig. 1.

### Characteristics of participating pharmacies and patients

Data for patients and pharmacies in the study are shown in Table 1. 61 pharmacists took part in 61 pharmacies. The survey was close to nationwide coverage (16 of 20 counties). Most of the pharmacies were in the capital (35.6%). 606 patients participated in the project (9.9 patients/pharmacy; SD = 3.0), 57.3% were women and 42.7% were males. 497 patients (mean = 8.1; SD = 2.2) took part in every requested meeting with the pharmacist (traced patients), 18.0% of the patients left the project. However, we used data from all the patients involved, not only from traced patients. The average age of patients was 65.0 years (SD = 11.9). 55.6% of the participants took ACE inhibitor and NSAID simultaneously, 39.8% of them took vitamin K antagonists, and 4.6% of them were included in both categories.

**Table 1:** Data for patients and pharmacists involved in the study (SD = standard deviation)

	∑	MEAN ± SD	Number of pharmacists involved:	61
Number of patients involved:	606	9.9 ± 3.0		
Traced patients:	497	8.1 ± 2.2	Number of pharmacies involved:	61
Dropout rate:	18.0%			
			Location of pharmacies (*n* = 59)	
Sex (*n* = 514)			Capital city:	35.6%
Male:	42.7%		County towns:	15.3%
Female:	57.3%		Other cities:	33.9%
			Other:	15.2%
Age of patients (*n* = 540)				
Mean ± SD (year)	65.0 ± 11.9		Distribution of patients (*n* = 526)	
Range:	24–96 year		Capital city:	34.0%
< 65 years:	50.6%		County towns:	15.2%
≥ 65 years:	49.4%		Other cities:	35.8%
			Other:	15.0%
Patient group (*n* = 540)				
ACEI-NSAID:	55.6%		Products (*n* = 540)	MEAN ± SD
VKA:	39.8%		Number of products:	7.9 ± 3.1
Both:	4.6%		Prescription drug:	6.3 ± 2.8
			OTC:	1.1 ± 1.1
			Other:	0.4 ± 0.8

### Medication review

Pharmacists have been conducting medication review at each consultation; they looked at medication taken from the point of necessity, effectiveness and safety. The medication review and the classification of drug-related problems were performed according to the Third Consensus of Granada on Drug-Related Problems classification system [21] and to the Hungarian National Committee of Pharmaceutical Care Metabolic Syndrome Pharmaceutical Care Programme. [26] In the process of assessing drug-related problems, the pharmacist classified the DRPs into six classes and identified the root cause. (Table 2).

**Table 2:** DRP classification and their underlying cause. [21, 26]

	Drug-related problem		Underlying cause
Necessity	DRP1	Untreated health problem. The patient suffers from a health problem as a consequence of not receiving the medicine that he/she needs.	Medication is necessary (lack of the required medication)
	DRP2	Effect of unnecessary medicine. The patient suffers from a health problem as a consequence of receiving the medicine that he/she does not need.	Unnecessary taken drug
			Multiple drug use from the same pharmacological category
Effectiveness	DRP3	Non-quantitative ineffectiveness. The patient suffers from a health problem associated with a non-quantitative ineffectiveness of the medication.	Improper medication choice
			Non-adherence
	DRP4	Quantitative ineffectiveness. The patient suffers from a health problem associated with a quantitative ineffectiveness of the medication.	Improper dosage
Safety	DRP5	Non-quantitative safety problem. The patient suffers from a health problem associated with a non-quantitative safety problem of the medication.	Interaction
			Side effects
	DRP6	Quantitative safety problem. The patient suffers from a health problem associated with a quantitative safety problem of the medication	Improper dosage
			Other

The number and cause of the drug-related problem were recorded by the pharmacist. In addition, the intervention was also recorded. The medication review was performed at all pharmacist meetings. Anonymous patient data were statistically analyzed.

### Statistical analysis

Statistical calculations were performed using SPSS 20.0. After the descriptive statistical analysis, two sample t test, paired sample t test, and a variance analysis were performed on continuous data to detect differences and correlations. When calculating the Pearson correlation coefficient, the p value for the correlation coefficient was < 0.005. For discrete data, the Kruskal-Wallis test and chi-square test were used. Control of normality was performed with Kolmogorov Smirnov test. The significance level was p = 0.05.

### Ethics approval and consent to participate

The project was implemented with the support and cooperation of the National Health Development Institute’s Primary Care Directorate [27]. The unified professional protocol made available in the course of the co-operation is a document agreed with the Primary Service Directorate. We have not received a waiver of ethics approval since the participation in the questionnaire survey, and the pharmaceutical service was not linked to one Institute (University) and was absolutely free and undoubtedly noninvasive, so IRB deemed unnecessary according to the similar national regulations. In Hungary according to Regulation No 44/2004 MoHSFA and Act XLVII of 1997, pharmacies did not need to be individually ethically licensed, because the service complies with statutory regulations, and pharmacies are legally entitled to perform such activities [28–31]. Verbal informed consent was obtained from all participants in the pharmacies; no written consent was required according to the Act CLIV of 1997 on Health (noninvasive pharmaceutical service and questionnaire survey) [32].

The investigation was a free service of pharmacies with operating licenses. The patients involved voluntarily participated in the process. Patients participating in the project received verbal information in accordance with the national regulations mentioned above. Qualified pharmacists conducted the project. The data were handled by pharmacy and health data management according to Act XLVII of 1997. Data were transmitted without personal information to process the results. The personal and health data of the patients included in the study were not damaged.

## Results

### Descriptive results

In the assessment of drug-related problems, 540 patients from 606 patients were collected and analyzed. On average, patients consumed 7.9 ± 3.1 medications and other products: 6.3 were prescription drug (SD = 2.8), 1.1 OTC (SD = 1.1) and 0.4 other product, for example dietary supplements (SD = 0.8). (Table 1).

**Fig. 2:**
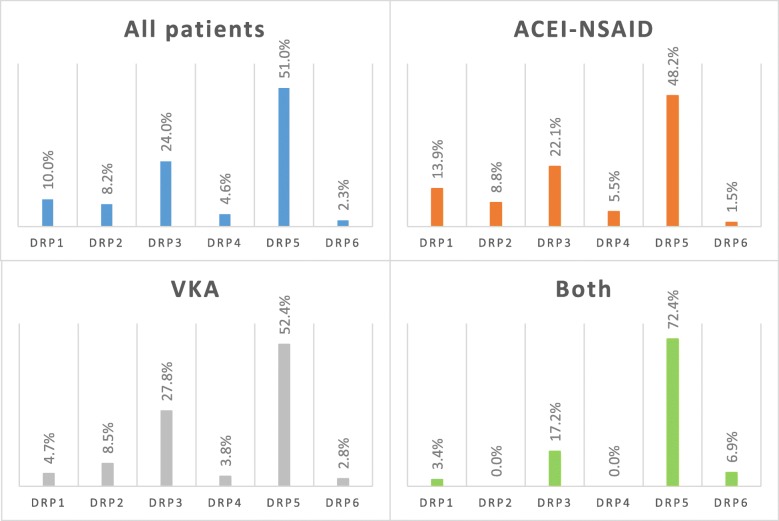
The relative proportion of different drug-related problem categories per patient group (All patients: all the participating patients (*n* = 571); ACEI-NSAID: patients taking ACE inhibitor and NSAID simultaneously (*n* = 330); VKA: patients taking vitamin K antagonist (*n* = 212); Both: patients included in both categories (*n* = 29); DRP: drug-related problem (see Table 2))

During the study, 571 drug-related problems were detected in these 540 patients, averaging 1.06 DRP per patient (SD = 1.07). The highest frequency category was DRP5 (non-quantitative safety problem: 51.0%). Approximately one-fourth of cases (24.0%) belonged to DRP3 (non-quantitative ineffectiveness) and 10% to DRP1 (untreated health problem). DRP2 (Effect of unnecessary medicine), DRP4 (Quantitative ineffectiveness) and DRP6 (Quantitative safety problem) were less frequent (8.2, 4.6, 2.3%). (Fig. 2-All patients).

**Fig. 3:**
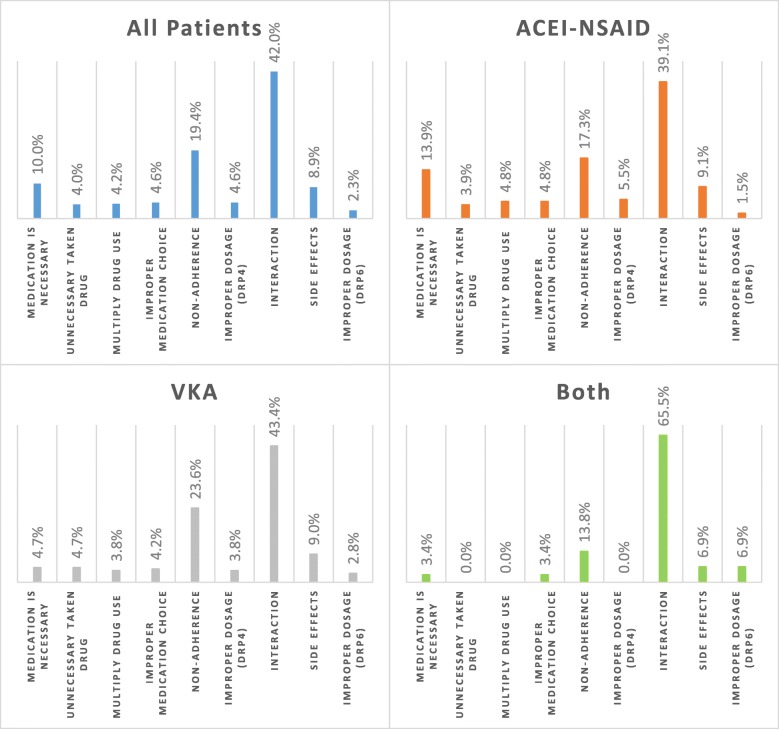
The relative proportion of the underlying cause of drug-related problems per patient group (All patients: all the participating patients (*n* = 571); ACEI-NSAID: patients taking ACE inhibitor and NSAID simultaneously (*n* = 330); VKA: patients taking vitamin K antagonist (*n* = 212); Both: patients included in both categories (*n* = 29); DRP: drug-related problem (see Table 2))

Analyzing the root causes of drug-related problems, the most common was the interaction (42.0%), the second was non-adherence (19.4%). The Quantitative safety problem caused by improper dosage was the rarest (2.3%). (Fig. 3*-All patients*).

In the case of ACEI-NSAID patients, the DRP1 category appears to be higher (13.9%) than in the case of VKA patients (4.7%). The ratio was reversed in the case of DRP3 (22.1 and 27.8%) and DRP5 (48.2% or 52.4%), the latter is due to a higher rate of interactions. (Figs. 2 and 3) The ratio of interaction was extremely high for those patients who were in both categories (65.5%). (Fig. 3) However, these differences are not significant either in the number of drug problems or in the occurrence of the individual categories and causes. There was no “other” problem that cannot be categorized elsewhere.

### Results of statistical analysis

There are no differences in the prevalence of drug-related problems between men and women (p = 0.070) and between the patients over and under 65 years. (p = 0.552).

There is a significant difference between the types of settlement in the occurrence of the drug-related problem. In the capital city, the pharmacists have found two DRPs per patient in a significantly higher ratio, while in other settlements it was markedly higher that the pharmacist found no mistake in the medication. There is a correlation between the number of DRPs and the total number of used medications, but the correlation is weak (Pearson correlation coefficient = 0.214 (p < 0.005)). The relationship between the number of prescription drugs and the number of drug-related problems is similar, somewhat lower (Pearson correlation coefficient = 0.152 (p < 0.005)).

Table 3 summarizes the rates of interventions used to eliminate drug-related problems. The most common intervention for the elimination of each underlying cause was indicated with bold number, while underlined number indicates the interventions with an incidence higher than 10%. Overall, the most commonly used intervention was education (25.4%) and medication replacement by the pharmacist (20.1%). More than 10% of the problems the intervention was not necessary (10.9%), or the pharmacist sends the patient to a physician (14.5%) or the pharmacist warned the GP (11.7%). The changing of the frequency and dosage in any direction were negligible.

**Table 3:** Drug-related problems and the ratio of the interventions used with each other for the total study population

DRP	Underlying cause	Interventions													∑
		Not necessary	Not happened	Change in frequency	Dose escalation	Dose reduction	Education	Helping with device	drug recommen- dation	Stop medication	Medication replacement	Sending to the doctor	Warning the GP	no data	
DRP1	Medication is necessary (lack of the required medication)	0.0%	0.0%	0.0%	0.0%	0.0%	1.8%	0.0%	**42.1%**	0.0%	1.8%	**42.1%**	12.3%	0.0%	10.0%
DRP2	Unnecessary taken drug	17.4%	0.0%	0.0%	0.0%	0.0%	8.7%	0.0%	0.0%	**43.5%**	4.3%	8.7%	13.0%	4.3%	4.0%
	Multiple drug use from the same pharmacological category	29.2%	0.0%	0.0%	0.0%	0.0%	0.0%	0.0%	0.0%	**50.0%**	4.2%	4.2%	12.5%	0.0%	4.2%
DRP3	Improper medication choice	3.8%	7.7%	0.0%	0.0%	0.0%	3.8%	0.0%	3.8%	0.0%	19.2%	**30.8%**	**30.8%**	0.0%	4.6%
	Non-adherence	0.0%	0.0%	0.0%	0.0%	0.0%	**82.0%**	8.1%	0.0%	0.0%	2.7%	1.8%	1.8%	3.6%	19.4%
DRP4	Improper dosage	3.8%	3.8%	0.0%	3.8%	0.0%	15.4%	0.0%	3.8%	0.0%	3.8%	30.8%	**34.6%**	0.0%	4.6%
DRP5	Interaction	18.8%	0.4%	0.0%	0.0%	0.0%	17.1%	0.0%	0.0%	5.0%	**39.2%**	5.8%	10.8%	2.9%	42.0%
	Side effects	7.8%	3.9%	0.0%	0.0%	0.0%	5.9%	0.0%	9.8%	3.9%	17.6%	**37.3%**	13.7%	0.0%	8.9%
DRP6	Improper dosage	0.0%	0.0%	7.7%	0.0%	23.1%	15.4%	0.0%	0.0%	0.0%	0.0%	**38.5%**	15.4%	0.0%	2.3%
	Other	–	–	–	–	–	–	–	–	–	–	–	–	–	**–**
∑		10.9%	1.1%	0.2%	0.2%	0.5%	**25.4%**	1.6%	5.4%	6.3%	20.1%	14.5%	11.7%	2.1%	100.0%

(DRP: drug-related problem; **Bold number**: the most common intervention for the elimination of each underlying cause; Underlined number: the interventions for each underlying cause with an incidence higher than 10% (*n* = 571))

## Discussion

Due to a large number of patients involved and the low drop-out rate, patients are interested and find the service provided by pharmacists useful. The fact that a large number of patients who had NSAIDs with an ACE inhibitor were included in the study underlined the relevance of this problem. Such a problem frequently does not show up at the doctor but at the pharmacy. The project involved a large number of patients with more than 5 medicines (also known as polypharmacy patients [33]).

It is noteworthy that patients use an average of 1 OTC drug on a regular basis, and that 4 out of 10 patients also use some other formulations (e.g. dietary supplements).

The use of these two product categories can only be supervised by the pharmacist. The patient’s medication is fully matched at expedition at the pharmacy only (Rx, OTC, other products) so the pharmacy service presented in the project plays an essential role in the assessment and resolution of drug-related problems. It is supported by a large number of DRPs that have been identified and classified in this project based on a drug-related problem classification system that has been used for the first time in Hungary. In addition to the reasons mentioned above, the overload of general practitioner services can also contribute. Among the DRP categories, there is a high amount of non-quantitative safety problems in all patient groups, which are mainly drug-drug or drug-other product interactions. The latter is also influenced by the patient’s involvement in the ACEI-NSAID group. However, we cannot talk about such a factor in the VKA group. This phenomenon is due to the uncontrolled use of the vast amounts of prescription and OTC medicines mentioned earlier and the other medicines are taken by 4 out of 10 patients. The problem may be solved by pharmacists who have resolved the situation in our research with education, medication replacement (especially OTC-OTC drug switching) and by sending the patient to the GP. In the case of interactions, “stop medication” has hardly occurred, and pharmacists seem to be hesitant to take this step, as they think that the physician is the one who competent to make this decision. Another major problem is the Non-quantitative ineffectiveness of the medication of a quarter of patients due primarily to their deliberate or unintended non-adherence.

Non-adherence is a widespread problem with chronic diseases for example in the case of conditions treated with ACE inhibitor and vitamin K antagonist. The pharmacist can help by detecting the problem and education. It is also important to mention that every tenth ACEI-NSAID patient is suffering from an untreated health problem. The research has shown that in many cases the pharmacist has noticed such a health problem, which has been solved by drug recommendation and by sending the patient to the GP. Based on these results, a medication review in the framework of basic pharmaceutical care can be a solution beyond the problems mentioned above in preventing the risks of self-medication.

In the case of medication review, it is also necessary to address the issue of competency conflict between the pharmacist and the general practitioner. By looking at the pharmacists’ interventions to resolve drug-related problems, we can see that 59.7% of the problems have been solved by the pharmacist without the involvement of a physician, primarily through education and the exchange of a patient’s drug with an OTC drug. The pharmacist in his/her own solved only about 5–6% of the cases by recommending a new drug or stopping a therapy, while the changing of frequency and dosage were as rare as possible without consultation with the physician. Pharmacists sent patients more to the physician without indicating the problem being diagnosed to them. Pharmacists preferred to send patients to doctors without consulting the GP, suggesting low levels of co-operation between two professions and pharmacists’ fears of doctors. Analyzing the effect of certain population factors on drug-related problems, it can be stated that among the examined factors, the number of DRPs is only influenced by the geographical location of the pharmacies. This, assuming that patients seek indirectly the pharmacy closest to their home first, refers indirectly to the influence of the type of residential township. Based on the results, it is assumed that a more extensive settlement poses a higher risk for patients, due to the less personal physician-patient and pharmacist-patient relationship, the more likely to be accessed by more accessible medical services. So the development of a regular pharmacist-patient relationship is of the utmost importance in this area.

## Conclusion

Based on the results of the 540 patients surveyed in the 61 Hungarian pharmacies we can conclude that patients are struggling with many drug-related problems that can be assessed and categorized by this system and which remain unrecognizable without pharmacists. To achieve this, further projects need to be developed to assist in the development of physician-pharmacist cooperation and the widespread dissemination of pharmaceutical care. Our results provide a reasonable basis for the widespread use of medication review. In the future, it would be worthwhile extending the study to other patient groups, such as elderly patients with polypharmacy.

## Abbreviations

ACEI-NSAID: Patients taking ACE inhibitor and NSAID simultaneously
DRP: Drug-related problem
VKA: Patients taking vitamin K antagonist
